# Antenatal Care as a Strategic Window for Long-Term Cardiovascular Risk Assessment

**DOI:** 10.1016/j.jacadv.2026.102962

**Published:** 2026-07-06

**Authors:** Debora Ehrhardt, Jonas Salm, Ralf Dechend, Michael C. Honigberg, Ulrich Pecks, Diana Lindner, Marianne Skovsager Andersen, Dirk Westermann, Janis M. Nolde, Lucas Bacmeister

**Affiliations:** a Clinic for Cardiology and Angiology, University Heart Center Freiburg – Bad Krozingen, University Medical Center Freiburg and Faculty of Medicine, University of Freiburg, Freiburg, Germany; b Experimental and Clinical Research Center, A Cooperation Between the Max-Delbrück-Center for Molecular Medicine in the Helmholtz Association and the Charité - Universitätsmedizin Berlin and HELIOS Clinic Berlin-Buch, Berlin, Germany; c Department of Medicine, Harvard Medical School, Boston, Massachusetts, USA; d Cardiovascular Research Center, Massachusetts General Hospital, Boston, Massachusetts, USA; e Department of Gynecology and Obstetrics, University Hospital of Wuerzburg, Wuerzburg, Germany; f Institute for Midwifery, Medical Faculty of the Julius-Maximilians-University and University Hospital of Wuerzburg, Wuerzburg, Germany; g Department of Endocrinology, Odense University Hospital, Denmark and Institute for Clinical Research, Faculty of Health Sciences, University of Southern Denmark, Odense, Denmark; h Department of Nephrology, University Medical Center Freiburg and Faculty of Medicine, University of Freiburg, Freiburg, Germany

**Keywords:** antenatal care, angiogenic markers, blood pressure, cardiac markers, cardiovascular risk, hypertensive disorders of pregnancy

## Abstract

Cardiovascular (CV) disease remains the leading cause of death in women, yet risk is often recognized late. Pregnancy is a physiologically demanding, routinely monitored period with repeated health care contact, standardized clinical assessments and blood-based testing, creating a strategic window to capture early signals of CV vulnerability long before conventional midlife risk assessment. Research has largely focused on hypertensive pregnancy complications, but most future CV events occur in women without such complications. Emerging evidence indicates that blood pressure trajectories, angiogenic and cardiac biomarkers, and routinely assessed metabolic parameters are associated with long-term maternal hypertension, subclinical CV phenotypes, and clinical CV events, even in the absence of overt hypertensive pregnancy complications. This review summarizes data on soluble fms-like tyrosine kinase-1, placental growth factor, natriuretic peptides, cardiac troponins, blood pressure, and the metabolic parameters weight and glycemic status, and discusses limitations of the current evidence base and key priorities for translating pregnancy-derived signals into clinically actionable CV risk assessment.

## Highlights

• Pregnancy offers a unique, routinely monitored window for early CV risk assessment in women.
• Hypertensive disorders of pregnancy affect ∼10% of pregnancies and carry future CV risk but limited population-level stratification utility.
• Pregnancy-derived biomarkers and clinical measures associate with long-term CV risk beyond hypertensive pregnancy complications.
• Prospective studies and prediction model validation are needed to translate pregnancy-derived signals into clinical use.

Cardiovascular (CV) disease remains the leading cause of death in women, accounting for approximately one-third of female mortality worldwide.^1^ Despite this burden, CV risk in women is frequently under-recognized, with risk often identified late in the disease course and diagnostic and preventive strategies historically derived from male-dominated cohorts.^2^, ^3^, ^4^ Earlier and more sex-specific identification of CV vulnerability in women therefore remains a critical unmet need.

Pregnancy represents a common and physiologically demanding life event characterized by profound CV and metabolic stress. Importantly, it is also a period of routine and repeated health care contact during which clinical and blood-based parameters are routinely assessed. As such, pregnancy offers a unique and underutilized window into long-term CV risk in women.^2^

To date, much of the research linking pregnancy and later CV disease has focused on adverse pregnancy outcomes (APOs), particularly hypertensive disorders of pregnancy (HDPs). Large-scale epidemiological studies have demonstrated that HDPs are independent predictors of premature CV events, conferring approximately a 2-fold increased relative risk.^5^, ^6^, ^7^, ^8^, ^9^ However, the modest relative risk and low prevalence of HDPs, affecting approximately 10% of pregnancies, substantially limit their population-attributable contribution to CV risk (Central Illustration).^10^ For instance, in the largest study to date examining the long-term CV impact of preeclampsia as an example, more than 95% of CV events occurred in women without a history of preeclampsia.^5^ Consistent with this, adding a history of HDPs to established CV risk scores has yielded only modest to negligible improvements in predicting CV risk in midlife women.^11^, ^12^, ^13^, ^14^, ^15^

**Central Illustration:**
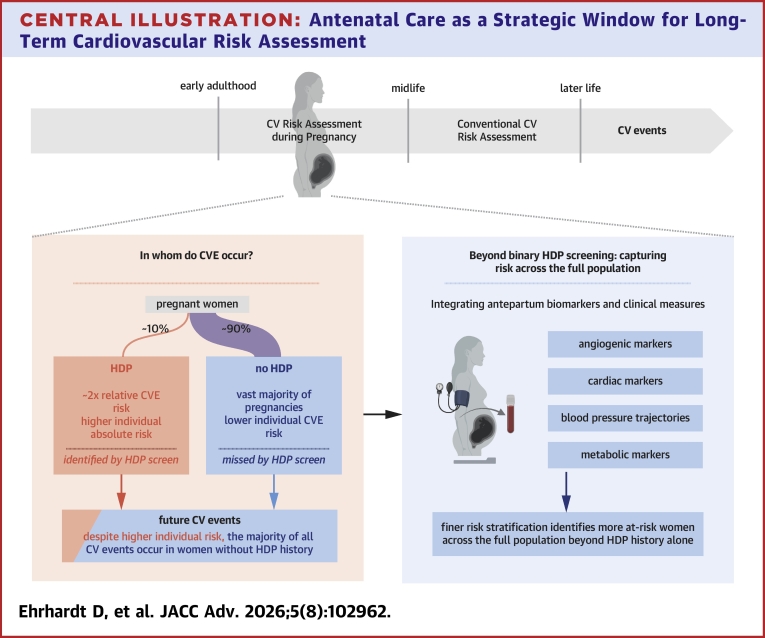
**Antenatal Care as a Strategic Window for Long-Term Cardiovascular Risk Assessment** Top: Pregnancy represents an opportunity for cardiovascular risk assessment earlier than conventional midlife screening, potentially identifying at-risk women decades before events occur. Bottom left: Hypertensive disorders of pregnancy (HDP) affect ∼10% of pregnancies and confer an approximately twofold higher relative risk of future cardiovascular events (CVE). However, since most pregnancies are unaffected, the majority of population-level CVE occur in women without HDP history, highlighting the limitation of HDP as a binary screening tool for long-term risk stratification. Bottom right: Incorporating antepartum biomarkers and clinical measurements including angiogenic markers, cardiac markers, blood pressure trajectories, and metabolic markers into pregnancy-based CV risk assessment may enable finer risk stratification beyond HDP history alone, capturing at-risk women across the broader population. CV = cardiovascular.

These findings highlight the limitations of relying on binary pregnancy outcomes alone for CV risk stratification. If pregnancy is to be leveraged as a screening window for future CV health, there is a need to move beyond clinical diagnoses and include biological signals that may reveal latent risk in the broad obstetric population. Biomarkers and clinical parameters measured during pregnancy represent such candidates, with the potential to capture subclinical CV vulnerability that remains invisible within entirely outcome-based frameworks (Central Illustration).

In this review, we synthesize the current, albeit still limited, evidence linking cardiac and angiogenic biomarkers, blood pressure measures, and metabolic parameters already assessed during antenatal care to long-term maternal CV risk. We focused on studies assessing: 1) angiogenic imbalance markers, specifically soluble fms-like tyrosine kinase-1 (sFlt-1) and placental growth factor (PlGF); 2) cardiac biomarkers including N-terminal pro-B-type natriuretic peptide (NT-proBNP) and cardiac troponins (cTns); 3) blood pressure levels and longitudinal patterns across gestation; and 4) the metabolic parameters weight and glycemic status. We included only studies in which indicators were measured antepartum, before delivery, and CV outcomes were assessed beyond 1 year postpartum to better separate long-term risk from transient pregnancy-related effects. Finally, we discuss biologically informed and clinically scalable approaches needed to leverage routine antenatal care as a window for earlier CV risk identification.

## Main

**Figure 1:**
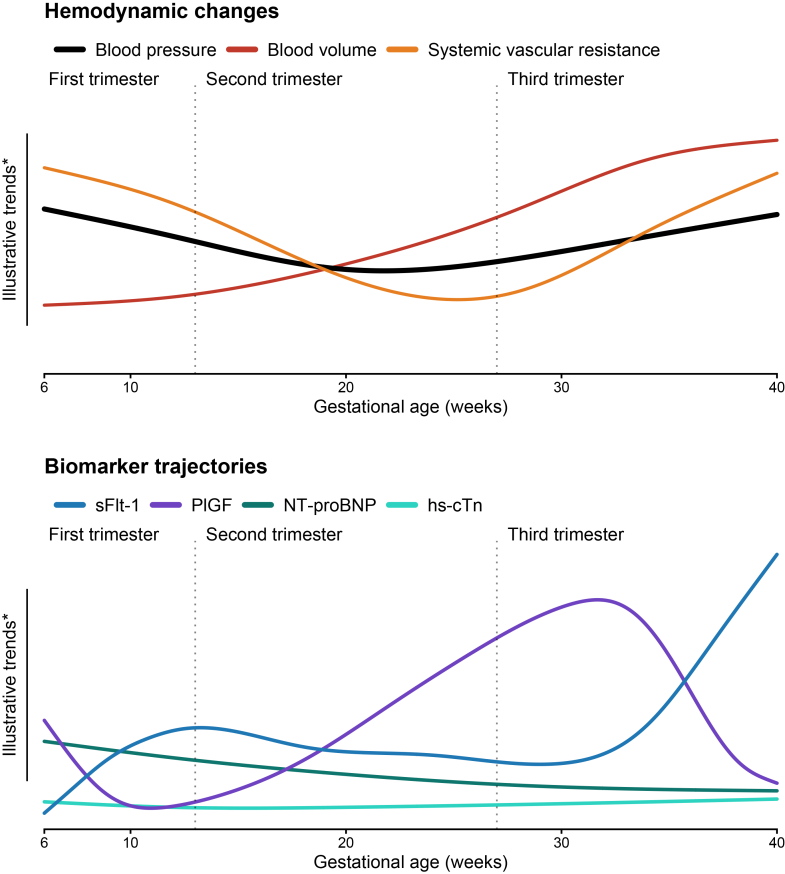
**Schematic Trajectories of Selected Cardiovascular Parameters During Normal Pregnancy** ∗Not to scale. The trajectories are based on physiological patterns reported previously.^16^, ^17^, ^18^, ^19^ hs-cTn = high-sensitivity cardiac troponin; NT-proBNP = N-terminal pro-B-type natriuretic peptide; PlGF = placental growth factor; sFlt = soluble fms-like tyrosine kinase.

**Figure 2:**
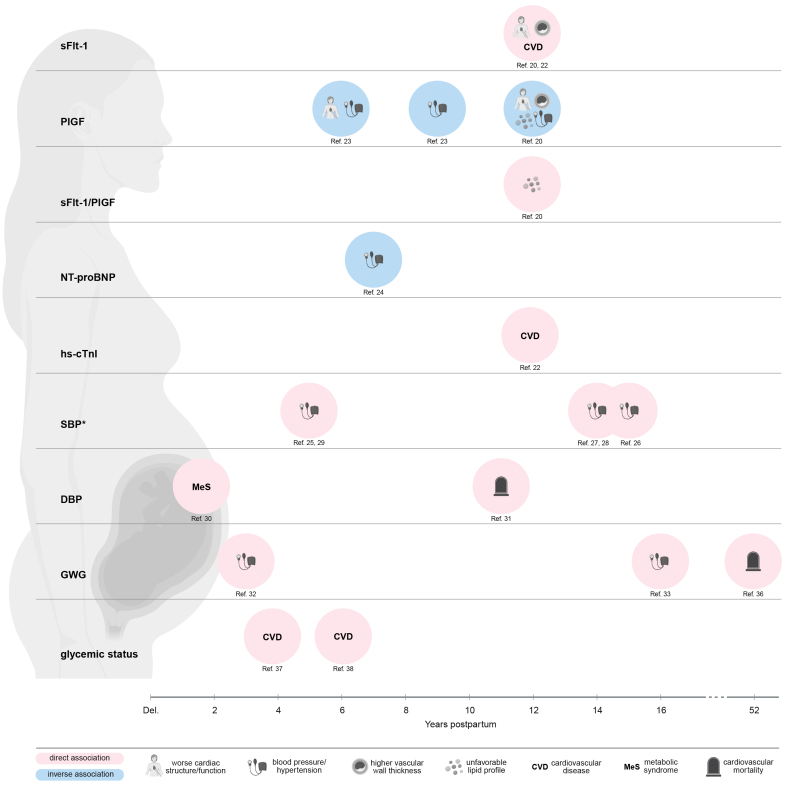
**Evidence Linking Antenatal Care Parameters to Long-Term Cardiovascular Risk** Each row represents one biomarker measured during pregnancy. The horizontal axis indicates years after delivery (Del.). Bubbles are positioned at the follow-up time point at which a statistically significant association was reported (median or mean where available, or the longest time point when only a range was provided). The icons within each bubble indicate the outcome type (see icon key). Bubble color reflects the direction of association for each marker: red = direct association; blue = inverse association. Reference numbers beneath each bubble indicate the source study; bubbles sharing a reference number originate from the same cohort. ∗Studies reporting systolic blood pressure only or combined blood pressure (SBP/DBP). CVD = cardiovascular disease; DBP = diastolic blood pressure; GWG = gestational weight gain; SBP = systolic blood pressure; sFlt-1/PlGF = ratio of sFlt-1 to PlGF; other abbreviations as in Figure 1.

To provide a quick overview, Figure 1 summarizes the typical gestational trajectories of key hemodynamic parameters and angiogenic and cardiac biomarkers in uncomplicated pregnancy. The Main section is then structured by biomarker, first outlining biological role and gestational patterns, then summarizing associations with HDPs, before reviewing evidence linking pregnancy measures to later CV outcomes. Table 1 and Figure 2 synthesize these associations in detail.

**Table 1:** Associations of Antepartum Biomarkers and Clinical Measures With Postpartum Cardiovascular Outcomes

Antepartum Biomarker				Association With Postpartum Cardiovascular Outcomes																				
Time Points During Gestation				1 to <10 y									≥10 y											
1st	2nd	3rd	At HDP	AOD	LAD	LV mass	HTN	MeS	CVD	FS	PWV	CRAE/CRVE	LV PWT	HDL	ApoA-1	cIMT	GLS	HTN	CeVD	CKD	DM	MACE	CVD	Death
			sFlt-1										^20^	^20^	^20^	^20^								
		sFlt-1																^21^	^21^	^21^	^21^	^21^		
		sFlt-1																					^22^	
			PlGF											^20^		^20^	^20^	∗^20^						
		PlGF																^21^	^21^	^21^	^21^	^21^		
		PlGF																					^22^	
	PlGF			^23^	^23^	^23^	∗^23^			^23^	^23^	^23^												
PlGF				^23^	^23^	^23^	∗^23^			^23^	^23^	^23^												
			sFlt-1/PlGF											^20^										
		sFlt-1/PlGF																^21^	^21^	^21^	^21^	^21^		
		hs-cTnI																					^22^	
		NT-proBNP																					^22^	
NT-proBNP							^24^																	
SBP							^25^																^22^	
	SBP																	^26^						
		SBP					^25^											^27^					^22^	
SBP trajectory																		^28^						
SBP trajectory							^29^																	
		DBP						^30^															^22^	
DBP																							^22^	
DBP trajectory								^30^																
DBP																								^31^
GWG							∗^32^																	
	GWG						∗^32^											∗^33^						
		GWG					∗^32^																	
Total GWG							∗^34^											∗^35^						^36^
	Glucose								^37^															
Glucose									^38^															

Associations between gestational biomarkers and cardiovascular risk parameters assessed postpartum. Outcomes are categorized by the postpartum time window (1-9.9 or ≥10 y) in which they were evaluated across studies. Symbols ( = direct association;  = inverse association;  = no significant association) reflect significance of adjusted effect estimates; adjustment covariates differed across studies. ∗Association with blood pressure (systolic, diastolic, or mean arterial) without a hypertension diagnosis.

ApoA-1 = apolipoprotein A-1; AOD = aortic root diameter; CeVD = cerebrovascular disease; cIMT = carotid intima-media thickness; CKD = chronic kidney disease; CRAE/CRVE = central retinal arteriolar/venular equivalent; CVD = cardiovascular disease, defined in the study referenced; DBP = diastolic blood pressure; DM = diabetes mellitus; FS = fractional shortening; GLS = global longitudinal strain; GWG = gestational weight gain; HDL = high-density lipoprotein; HDP = hypertensive disorder of pregnancy; hs-cTnI = high-sensitivity cardiac troponin I; HTN = hypertension; LAD = left atrial diameter; LV mass = left ventricular mass; LVPWT = left ventricular posterior wall thickness; MACE = major adverse cardiovascular events; MeS = metabolic syndrome; NT-proBNP = N-terminal pro-B-type natriuretic peptide; PlGF = placental growth factor; PWV = pulse wave velocity; SBP = systolic blood pressure; sFlt-1 = soluble fms-like tyrosine kinase-1; sFlt-1/PlGF = ratio of sFlt-1 to PlGF.

### Angiogenic biomarkers

#### Biological role and gestational trajectories

sFlt-1 is an antiangiogenic factor that binds vascular endothelial growth factor and PlGF, limiting their interaction with endothelial receptors and thereby promoting an antiangiogenic milieu and endothelial dysfunction. In uncomplicated pregnancy, circulating sFlt-1 levels remain low in early and mid-gestation and rise in the third trimester, peaking near term before declining postpartum.^39^, ^40^, ^41^, ^42^

PlGF is a proangiogenic, primarily placenta-derived factor that supports placental vascular development. Levels increase from the first trimester, peak in late second/early third trimester, and decline toward term.^39^, ^40^, ^41^, ^42^, ^43^, ^44^, ^45^

Given their pronounced gestational age dependency, standardized sampling time points and gestational age-specific reference ranges are essential for interpretability in pregnancy-based risk phenotyping.

#### Association with HDPs and clinical utility inside pregnancy

In HDPs, particularly preeclampsia, sFlt-1 concentrations are commonly elevated in late pregnancy, often weeks before clinical onset, and correlate with disease severity.^41^^,^^42^^,^^46^ In contrast, PlGF levels appear reduced, likely reflecting both decreased placental expression and sequestration by excess sFlt-1.^41^^,^^42^^,^^47^ Accordingly, the sFlt-1/PlGF ratio is used clinically to support the diagnostic evaluation of suspected preeclampsia,^42^^,^^48^^,^^49^ and has been shown to improve diagnostic performance compared with either marker alone.^42^^,^^48^, ^49^, ^50^

#### Pregnancy biomarker levels and their association with postpartum CV outcomes

Beyond their role in pregnancy complications, several studies have examined whether antepartum (anti-)angiogenic profiles are associated with maternal CV health in the years following pregnancy.

In a prospective cohort of 5,475 women, Benschop et al^23^ assessed early and mid-pregnancy PlGF levels and evaluated CV phenotypes up to 9 years postpartum. At 6 years, outcomes included maternal blood pressure, cardiac structure, arterial stiffness, and retinal vessel calibers, with blood pressure reassessed at 9 years. Mid-pregnancy PlGF was inversely associated with aortic root diameter, left atrial diameter, left ventricular mass, and systolic blood pressure. Early pregnancy PlGF also showed similar inverse associations with aortic diameter and systolic blood pressure at 6 years. No associations were observed with fractional shortening, arterial stiffness, or retinal vessel calibers. Importantly, the inverse association with later cardiac structural measures and with systolic blood pressure persisted after excluding women with pregnancy complications, suggesting an independent association of angiogenic markers with later subclinical CV phenotypes, even in the absence of overt HDP status.^23^

Recent evidence extends these findings from subclinical phenotypes to clinical endpoints. Data from a cohort including 2,056 women with biomarker measurements obtained during pregnancy further support a potential link between pregnancy-derived angiogenic signals and later clinical outcomes. Higher third-trimester sFlt-1 concentrations were associated with an increased risk of subsequent CV disease over a median follow-up of 11.9 years, independent of established clinical risk factors. In addition, incorporation of third-trimester sFlt-1 levels improved discrimination for later CV disease beyond age alone, whereas a clinical model including age, systolic blood pressure and non–high-density lipoprotein (HDL) cholesterol did not yield comparable improvement.^22^

In smaller studies sampling angiogenic markers at the time of HDP diagnosis or clinical suspicion, findings have been mixed: Garrido-Gimenez et al^20^ evaluated in a prospective study whether antepartum angiogenic marker levels, measured at HDP diagnosis, were associated with CV phenotypes up to 12 years postpartum. They followed 43 women with prior preeclampsia and 21 controls, assessing CV risk through hemodynamic measures, cardiac structure and function, lipid levels, and carotid intima-media thickness. sFlt-1 levels were positively associated with left ventricular posterior wall thickness and carotid intima-media thickness and inversely associated with apolipoprotein A1 levels. Both sFlt-1 and sFlt-1/PlGF ratios were linked to lower HDL. In contrast, higher PlGF levels were associated with a more favorable CV profile, including higher HDL, lower mean arterial blood pressure, reduced carotid intima-media thickness, and better cardiac function. These findings appeared also independent of preeclampsia status.

In contrast, in a likely highly underpowered retrospective cohort study of 117 women sFlt-1, PlGF, and their ratio, measured at the time of clinical suspicion of preeclampsia, were not associated with the incidence or prevalence of chronic kidney disease, diabetes mellitus, major adverse CV events, cerebrovascular disease, or hypertension 10 years after pregnancy.^21^

Preclinical models provide partial mechanistic support for a link between angiogenic imbalance and later CV vulnerability, although results vary by model and exposure. While some studies report no sustained postpartum changes in blood pressure or vascular function after gestational sFlt-1 exposure,^51^, ^52^, ^53^ 1 study observed exaggerated blood pressure responses to a high-salt diet and angiotensin II.^53^ Other models have also demonstrated persistent postpartum hypertension, endothelial dysfunction and cerebrovascular and neuroinflammatory alterations.^54^, ^55^, ^56^

#### Evidence outside pregnancy

Outside pregnancy, sFlt-1 and PlGF have been evaluated in acute ischemic syndromes, heart failure, and population-based cohorts, providing context for their interpretation and potential prognostic utility. Unless otherwise specified, the studies summarized in this paragraph were conducted in mixed-sex cohorts and women-only cohorts are explicitly noted. In acute and chronic CV disease settings, sFlt-1 is frequently elevated and has been linked to disease severity and adverse outcomes.^57^^,^^58^ In heart failure, sFlt-1 shows a more consistent association with severity and prognosis,^59^ with some studies suggesting improved risk stratification when assessed alongside natriuretic peptides.^60^^,^^61^ In a population-based cohort, higher sFlt-1 was associated with incident atherosclerotic CV disease events over long-term follow-up.^62^ PlGF appears more context-dependent: preclinical data support roles in postischemic angiogenesis and repair,^63^, ^64^, ^65^ yet PlGF has also been linked to inflammatory signaling and plaque formation,^66^^,^^67^ suggesting that PlGF may act less as a uniformly protective factor than as a context-dependent marker of vascular stress, remodeling, or repair. Clinical studies have reported associations of higher PlGF with worse outcomes in acute coronary syndromes.^68^^,^^69^ In initially healthy women, higher baseline PlGF was associated with subsequent risk of coronary heart disease over long-term follow-up,^70^ and PlGF levels 6 years after an index pregnancy were associated with higher subsequent blood pressure and increased risk of later hypertension.^71^ Taken together, evidence outside pregnancy suggests that these angiogenic factors can track CV pathology, but their incremental prognostic value beyond established biomarkers remains heterogeneous.^72^

#### Interpreting gestational levels of angiogenic markers in relation to later CV risk

sFlt-1 and PlGF change substantially over the course of pregnancy, so interpretation is relative to gestational age. For sFlt-1, first trimester concentrations have not been associated with adverse long-term clinical outcomes in available data,^22^ whereas these associations emerge when sFlt-1 is assessed later in gestation,^20^^,^^22^ consistent with its rise toward term and its link to an antiangiogenic, endothelial-stress milieu in HDP. Outside pregnancy, higher sFlt-1 has also been associated with adverse CV outcomes, providing context that late-gestation sFlt-1 may reflect broader endothelial vulnerability. Correspondingly, the most direct evidence for prediction of later clinical CV outcomes has been reported for third-trimester sFlt-1.^22^

PlGF appears more context and time dependent. During pregnancy, it is linked to placentation and uteroplacental vascular development, such that lower PlGF in early to mid-gestation may signal impaired placental vascular adaptation and/or reduced angiogenic reserve. Later in gestation, interpretation may differ as PlGF physiology changes and sFlt-1 rises (binding/sequestration). Against this background, associations reported for late-pregnancy PlGF and subsequent clinical outcomes have been less consistent,^22^ and the direction of associations observed outside pregnancy does not necessarily parallel those seen during pregnancy.

### Cardiac biomarkers

#### Biological role and gestational trajectories

Cardiac biomarkers provide complementary readouts of maternal CV adaptation. NT-proBNP is the inactive amino-terminal fragment of proBNP, the prohormone of BNP, released from cardiac myocytes in response to volume expansion and increased myocardial wall stress. During pregnancy, NT-proBNP is highest in the first trimester,^16^^,^^73^, ^74^, ^75^ then declines by mid-to-late pregnancy and approach levels comparable to those in nonpregnant women.^16^^,^^73^, ^74^, ^75^, ^76^ Around delivery, there is a transient early postpartum surge followed by a return toward baseline.^75^^,^^77^

Cardiac troponins I and T (cTnI and cTnT), are cardiac-specific biomarkers of myocardial injury that can be quantified using high-sensitivity (hs) assays (hs-cTnI, hs-cTnT). In uncomplicated pregnancy, hs-cTnI concentrations are typically very low and comparable to those in nonpregnant women, remaining largely stable across trimesters.^17^^,^^78^, ^79^, ^80^ However, in 1 cohort a modest upward trend across gestation has been reported.^81^

#### Association with HDPs and clinical utility inside pregnancy

In established preeclampsia, natriuretic peptides are typically elevated, consistent with increased cardiac strain and/or subclinical cardiac dysfunction at presentation, reflecting disease pathophysiology.^82^, ^83^, ^84^ In contrast, lower first-trimester NT-proBNP has been observed in women who later develop HDPs or APOs, suggesting altered early CV adaptation rather than overt cardiac strain.^24^^,^^85^ In the early third trimester, NT-proBNP shows a time-dependent association, with higher levels linked to imminent preeclampsia risk whereas lower levels are associated with higher risk beyond 4 weeks.^86^ Overall, natriuretic peptides have not yet shown consistent incremental value for HDP risk stratification beyond established predictors, particularly angiogenic markers.^86^

For markers of myocardial injury, elevated hs-cTnI levels have been associated with an increased risk of HDP,^78^^,^^81^ with the association appearing particularly pronounced among women who later develop preterm preeclampsia.^81^ Moreover, among women with HDP, elevated hs-cTnI levels have been associated with maternal complications.^87^

#### Pregnancy biomarker levels and their association with postpartum CV outcomes

Beyond short-term pregnancy complications, a smaller body of work has examined whether gestational cardiac biomarkers relate to postpartum hypertension, subclinical cardiac phenotypes, or later CV events.

Hauspurg et al^24^ evaluated 4,103 women from the noMoM2b (Nulliparous Pregnancy Outcomes Study: Monitoring Mothers-to-Be) Heart Health Study for the development of hypertension 2 to 7 years postpartum. Higher NT-proBNP levels measured in the first trimester were associated with a lower likelihood of developing hypertension after pregnancy. This association remained significant after adjustment for multiple confounders, including HDPs, suggesting that NT-proBNP may capture aspects of early-pregnancy CV physiology relevant to long-term maternal hypertension risk, also beyond HDP development.^24^

Data linking gestational cardiac biomarkers to clinical CV events remain limited. Recently, higher third-trimester hs-cTnI concentrations were independently associated with an increased risk of subsequent CV disease over a median follow-up of 11.9 years.^22^ However, unlike sFlt-1, adding hs-cTnI did not improve discrimination for later CV disease beyond age alone. Moreover, in the same study, third-trimester NT-proBNP was not independently associated with future CV events.

#### Evidence outside pregnancy

Outside pregnancy, natriuretic peptides are established biomarkers in heart failure and are recommended by major guidelines to support diagnosis and risk stratification.^88^^,^^89^ Beyond heart failure, higher natriuretic peptide concentrations have been consistently associated with later adverse CV outcomes.^90^, ^91^, ^92^, ^93^ In female primary-prevention cohorts, NT-proBNP has been independently associated with incident CV disease.^94^^,^^95^ Adding NT-proBNP to established risk models has yielded modest improvements in discrimination and reclassification in some populations, with inconsistent findings potentially reflecting differences in sample size and population characteristics.^94^^,^^95^

Cardiac troponins are central to the diagnosis of myocardial infarction, and high-sensitivity assays enable earlier rule in/rule out.^96^^,^^97^ Beyond myocardial infarction, higher baseline hs-cTnI is associated with subsequent coronary events, stroke, heart failure, and mortality,^98^, ^99^, ^100^ and relates to subclinical cardiac phenotypes.^101^ Adding hs-cTnI to conventional risk factors yields small but statistically significant improvements in discrimination and/or reclassification in primary-prevention risk models.^102^^,^^103^ Some sex-stratified analyses report stronger associations and/or better discrimination for hs-cTnI in women than in men, positioning it as a potentially useful marker for CV risk assessment during pregnancy.^104^^,^^105^

#### Interpreting gestational levels of cardiac markers in relation to later CV risk

Cardiac biomarkers provide readouts of maternal CV physiology during a period of substantial hemodynamic change, but their interpretability in pregnancy is marker and gestation dependent. For cTns, the direction of association with CV risk appears broadly aligned with nonpregnant populations,^98^, ^99^, ^100^, ^101^, ^102^, ^103^ with higher concentrations associated to CV disease,^22^ likely reflecting a shared underlying pathophysiology not specific to pregnancy. However, in a lower-risk population-based pregnancy cohort, third-trimester hs-cTnI concentrations did not add incremental predictive value beyond maternal age,^22^ although interpretation is limited by the high proportion of values below the limit of detection, and more sensitive assays or evaluation in higher-risk populations may produce different findings.

For natriuretic peptides, however, pregnancy context does modify interpretation: NT-proBNP levels are elevated at the time of HDP,^82^, ^83^, ^84^ consistent with cardiac stress at presentation, whereas lower NT-proBNP, particularly in early gestation, is associated with a higher risk of subsequent HDP^85^ and incident postpartum hypertension.^24^ However, in the only study conducted to date, no predictive value for future CV disease after pregnancy was apparent.^22^

### Blood pressure

#### Biological role and gestational trajectories

Blood pressure is a universal, low-cost component of antenatal surveillance. As an integrative measure of maternal hemodynamics, it reflects CV adaptation to pregnancy and may capture subclinical vascular susceptibility before overt disease, thereby identifying women at higher long-term risk.^106^ In uncomplicated pregnancy, blood pressure typically declines from early gestation to a mid-second trimester nadir, followed by a gradual rise toward term.^107^, ^108^, ^109^

#### Association with HDPs and clinical utility inside pregnancy

Blood pressure also underpins the clinical recognition of hypertensive pathology in pregnancy. It is central to the definition and classification of HDPs.^110^ Beyond its diagnostic role, early-pregnancy blood pressure levels and trajectories provide prognostic information: Elevated first-trimester blood pressure, even below traditional hypertensive thresholds, and an attenuated early-gestation decline are associated with increased risk of subsequent HDP.^28^^,^^111^, ^112^, ^113^ The mean arterial pressure in the first trimester shows moderate-to-good discrimination for gestational hypertension and preeclampsia,^114^ with improved performance when incorporated into multivariable screening models that combine maternal characteristics with biomarkers and clinical measures.^115^

#### Pregnancy measurements and their association with postpartum CV outcomes

Accumulating evidence indicates that pregnancy blood pressure also carries long-term CV relevance. Modest elevations in blood pressure during pregnancy, even within ranges traditionally considered normal, have been consistently associated with future CV risk. In a cohort of 667 women, moderately elevated blood pressure (defined as repeated systolic ≥120 mm Hg and/or diastolic ≥80 mm Hg) before 20 weeks’ gestation was strongly associated with an increased risk of developing hypertension 7 to 15 years postpartum.^26^ Similarly, in the ROLO (Randomized control trial of low glycemic index diet to prevent macrosomia) cohort, elevated blood pressure (120-129 mm Hg/<80 mm Hg) recorded at booking visit (13 weeks’ gestation) was modestly associated with increased odds of stage 2 hypertension 5 years postpartum. More robust associations were observed later in pregnancy, with elevated blood pressure at both 28 and 34 weeks’ gestation independently associated with a higher risk of stage 1 hypertension at 5-year follow-up.^25^ Brady et al^25^ further demonstrated that even small, incremental increases in blood pressure during late gestation carry long-term significance. These findings were further supported by data from over 4,500 women without chronic hypertension, in whom elevated blood pressure (120-129/<80 mm Hg) measured in the early third trimester was associated with a significantly increased risk of developing both stage 1 and stage 2 hypertension 10 to 14 years after pregnancy, compared to lower readings.^27^ In another cohort of 309 normotensive women, diastolic blood pressure ≥80 mm Hg in late gestation (≥37 weeks) was associated with increased odds of metabolic syndrome within the first 2 years postpartum, complementing trajectory-based findings from the same study.^30^

Beyond intermediate outcomes, blood pressure levels during pregnancy have also been associated with long-term CV mortality. In a national cohort of over 1.2 million women, the highest single diastolic blood pressure recorded during pregnancy stratified future mortality risk in a graded fashion, with progressively higher values associated with markedly greater CV death rates over more than a decade of follow-up in early midlife.^31^ However, when moving from intermediate outcomes and mortality to composite clinical CV disease, findings are less consistent: in the cohort evaluated by Bacmeister et al^22^, systolic and diastolic blood pressure at 12 and 29 weeks’ gestation were not independently associated with later composite CV disease. These discrepancies may reflect endpoint definition (death vs composite CV disease), low event rates, and heterogeneity in covariate adjustment, and underscore that the strength of association with hard CV disease outcomes remains incompletely defined.

In addition to single time-point measurements, longitudinal blood pressure trajectories across pregnancy may offer additive information. In a cohort of 359 normotensive women (<130/80 mm Hg), a high-normal systolic blood pressure trajectory with a sustained plateau throughout gestation and absence of the expected mid-pregnancy dip (“consistently elevated”) was strongly associated with incident hypertension 2 to 5 years postpartum. Notably, this association remained robust even after excluding individuals who developed gestational hypertension or preeclampsia.^29^ In a large population-based study of over 170,000 women, systolic blood pressure trajectories established before 20 weeks’ gestation were strongly associated with incident hypertension up to 14 years postpartum, with risk increasing progressively across trajectory groups. Women whose systolic blood pressure followed an elevated-stable trajectory (126-129 mm Hg) had the highest risk of hypertension, and this risk was even greater in those who also developed an HDP.^28^ Diastolic blood pressure trajectories have also been linked to postpartum cardiometabolic risk. In a cohort of 309 normotensive pregnancies, an elevated J-shaped diastolic blood pressure trajectory, characterized by higher diastolic blood pressure beginning in the first trimester and persisting through term, was strongly associated with postpartum metabolic syndrome within 2 years postpartum, compared with a low J-shaped reference trajectory.^30^

Taken together, evidence across cohorts consistently indicated that both cross-sectional blood pressure levels and longitudinal trajectories during pregnancy predict adverse cardiometabolic outcomes years later. Even moderate antepartum elevations well below diagnostic thresholds for HDPs are associated with an increased risk of future hypertension. Trajectory-based approaches may add further prognostic value, particularly when the expected mid-pregnancy dip is blunted or absent, suggesting latent risk that may not be apparent from conventional snapshot measurements.

#### Pregnancy blood pressure as an opportunistic risk signal

These observations are consistent with evidence outside of pregnancy, where blood pressure is an established, continuous determinant of CV risk, with graded associations across ranges traditionally considered normotensive.^116^, ^117^, ^118^ Within pregnancy, blood pressure differs from circulating biomarkers in that it is universally obtained, low cost, and repeatedly measured across gestation, with the possibility of home and ambulatory monitoring. The consistent observation that modest antepartum elevations and high-normal or flattened trajectories predict later hypertension and broader cardiometabolic risk suggests that pregnancy blood pressure can capture latent vascular susceptibility also in normotensive pregnancies and in the absence of HDPs.

On top of that, a distinct, unresolved question appears in the context of blood pressure as an opportunistic measure: Do blood pressure trajectories characteristic of pregnancy provide incremental predictive value for long-term CV risk beyond what conventional blood pressure monitoring across the life course already offers? In principle pregnancy could act as a natural hemodynamic CV “stress test”, in which the circulatory demands of gestation unmask latent vascular susceptibility, such that levels and trajectories observed during pregnancy might outperform, or add to, blood pressure measurements obtained before or after pregnancy, or in women who have never been pregnant. To date, however, existing studies have not systematically benchmarked gestational blood pressure patterns against nonpregnant blood pressure or established CV risk scores.

### Routinely assessed metabolic markers

Alongside the angiogenic and CV biomarkers discussed previously, antenatal care routinely includes 2 straightforward metabolic measurements: weight and glycemic status.

#### Gestational patterns and clinical assessment

Maternal weight is routinely assessed throughout pregnancy,^119^^,^^120^ with baseline body mass index (BMI) recorded at the first prenatal visit providing an initial cardiometabolic reference.^120^ Gestational weight gain (GWG) reflects the cumulative physiological adaptations of pregnancy, supporting fetal development through changes in maternal physiology and metabolism.^120^^,^^121^ In uncomplicated pregnancies, weight gain follows a nonlinear pattern: modest in first trimester, accelerating through the second and third trimester.^120^^,^^122^

Glucose is essential for fetal growth and development, driving a progressive metabolic shift in maternal physiology to ensure adequate fetal supply.^123^ As pregnancy advances, insulin resistance increases, which may exceed maternal compensatory capacity, resulting in gestational diabetes mellitus (GDM), the primary indication for glucose testing during pregnancy.^124^ In uncomplicated pregnancies, postload glucose concentrations rise progressively through the second and third trimester, reflecting increasing insulin resistance,^125^ and are assessed by the universal mid-pregnancy oral glucose tolerance test (OGTT).

#### Associations with HDPs and clinical utility inside pregnancy

Although pre-pregnancy obesity is a recognized risk factor incorporated into clinical guidelines for preeclampsia risk assessment,^126^^,^^127^ GWG is also independently associated with HDPs,^128^^,^^129^ with early-pregnancy GWG preceding the development of preeclampsia and gestational hypertension.^129^

Alongside to the association of GDM with subsequent preeclampsia,^130^^,^^131^ postload values from mid-pregnancy OGTT were independently associated with preeclampsia^132^ and the pattern of glucose response differentiated risk for early- vs late-onset HDP.^133^

#### Pregnancy measurements and postpartum CV outcomes

Growing evidence suggests that GWG and glucose levels similarly carry long-term CV relevance. Walter et al^32^ demonstrated in a cohort of 801 women that a greater first-trimester GWG rate, but not second- or third-trimester gain, was independently associated with higher systolic blood pressure at 3 years postpartum, a finding extended by Fraser et al^33^ to 16 years, where in a cohort of 2,356 women mid-pregnancy GWG in women with normal baseline BMI was significantly associated with higher systolic and diastolic blood pressure. Notably, studies examining total GWG per Institute of Medicine guidelines found no significant association with blood pressure at 8 and 17 years postpartum.^34^^,^^35^ Extending the follow-up horizon considerably, Hinkle et al. assessed total GWG in relation to mortality over a median of 52 years in 46,042 women. Excessive GWG was associated with increased CV and all-cause mortality in women with normal pre-pregnancy weight, and with CV mortality alone in women who were underweight before pregnancy. In women who were overweight before pregnancy, excessive GWG was associated with all-cause and diabetes-related mortality, whereas no significant associations were observed in women with pre-pregnancy obesity.^36^

Data linking glucose measurements independent of GDM diagnosis to later CV risk remains limited. In a population-based cohort of 259,164 women, each 1 mmol/L increment in the 1-hour 50g glucose challenge test results at 24 to 28 weeks was associated with a higher risk of CV disease over a median follow-up of 3.9 years, also independent of GDM and HDP. Notably, even women with results in the upper-normal range (7.2-7.7 mmol/L) had significantly elevated CV risk compared to those with results ≤7.1 mmol/L.^37^ Complementary evidence from a retrospective case-control study of 6,880 women showed that a glucose level >5.5 mmol/L during pregnancy was an independent predictor of hospitalization for CV and cerebrovascular disease over a mean follow-up of 6.2 years, after adjustment for GDM and HDP.^38^

#### Gestational weight and glycemic status as opportunistic CV risk signals

This evidence aligns with observations in the nonpregnant population, where the link between BMI and CV morbidity and mortality is firmly established. Like blood pressure, weight is easily obtained and repeatedly assessed throughout pregnancy. Pre-pregnancy BMI outside the normal range is associated with postpartum hypertension, CV events, and mortality.^134^, ^135^, ^136^ Emerging evidence also connects GWG to postpartum CV mortality.^36^ Yet GWG may be a crude signal as it conflates fetal and maternal components inseparable by scale alone.^119^ Maternal fat mass is itself a recognized cardiometabolic risk pathway,^137^ raising the question of what GWG captures beyond fat deposition during pregnancy. Trimester-specific trajectories may aid differentiation, as distinct growth periods contribute differently to total gain and deviations carry prognostic meaning,^32^^,^^119^ raising a further question of whether GWG patterns can flag women at heightened CV risk even within recommended ranges.

Dysglycemia is an established driver of vascular injury and CV disease in the general population, also operating below the diabetes threshold.^138^^,^^139^ Pregnancy offers an opportunity to capture this risk as glucose is assessed during antenatal care. Beyond GDM as an independent predictor of future CV disease,^140^^,^^141^ emerging evidence suggests that subdiagnostic hyperglycemia on the mid-pregnancy OGTT independently associates with long-term CV risk.^37^^,^^38^ Other glucose parameters measurable during pregnancy, like trimester-specific or trajectory-based fasting glucose or glycosylated hemoglobin concentrations, may carry additional prognostic information, yet remain largely unexamined in relation to long-term CV outcomes.

## Discussion

This review highlights emerging evidence that biological signals, including angiogenic and cardiac biomarkers, blood pressure, and metabolic markers, captured during pregnancy, are associated with later maternal CV risk. Across multiple cohorts with follow-up extending up to 50 years, pregnancy measurements have been linked to subsequent hypertension, adverse subclinical CV phenotypes, and clinical CV events. Several associations persisted after adjustment for HDPs, suggesting that routine antenatal measurements carry CV risk information across the obstetric population also beyond binary obstetric outcomes.

The marker classes reviewed reflect distinct domains of pregnancy physiology and pathology. Angiogenic markers show pregnancy-specific trajectories consistent with placental signaling and deviations may be consistent with placental dysfunction and downstream maternal endothelial stress.^46^^,^^142^^,^^143^ Cardiac biomarkers may capture complementary axes of CV adaptation and injury. Natriuretic peptides appear to reflect hemodynamic adaptation earlier in pregnancy and, later, myocardial wall stress, consistent with their gestational pattern and elevations observed in HDP phenotypes.^24^ By contrast, cTns more directly index myocardial injury and potentially reflect underlying CV susceptibility present before pregnancy.^81^ Viewed together, these markers could support a more biologically informed approach to capturing different aspects of CV stress during pregnancy with the potential to identify women at higher long-term CV risk.

Blood pressure differs from circulating biomarkers in that it is universally obtained, low cost, and repeatedly measured across gestation. Evidence is consistent that modest elevations during pregnancy, even below diagnostic thresholds for hypertensive disorders, predict later hypertension and broader cardiometabolic risk. Trajectory-based approaches add important nuances, particularly when the expected mid-pregnancy dip is attenuated or absent, suggesting latent vascular susceptibility that may not be apparent from snapshot measurements. This is conceptually aligned with nonpregnant populations, where blood pressure is a continuous determinant of CV risk, and it underscores the immediate scalability of pregnancy blood pressure phenotyping as a screening component.

Metabolic parameters, like GWG and glycemic status, add to that because they are like blood pressure, easy to obtain, and already part of antenatal care. Consistent with evidence in nonpregnant populations, elevated GWG and glycemic status reflect an unfavorable cardiometabolic profile, itself associated with heightened long-term CV risk, and may carry additional susceptibility information specific to the pregnancy period, as both have been independently linked to adverse CV outcomes.

### Evidence gaps and methodological barriers

Current evidence is constrained by several methodological limitations that restrict interpretability and slow translation. Many biomarker studies rely on single time-point measurements despite pronounced gestational-age dependence, which reduces comparability across cohorts and can obscure clinically relevant dynamics. Differences in exposure definition, sampling windows, assay platforms, preanalytical handling, batch effects, and covariate adjustment strategies further increase heterogeneity and may contribute to inconsistent findings across studies. For blood pressure, the literature more clearly demonstrates the value of longitudinal phenotyping, yet trajectory-based approaches for the other markers remain uncommon. Moreover, few studies have evaluated whether gestational levels or trajectories improve prediction beyond conventional blood pressure measurements obtained outside pregnancy. A further limitation is that most studies have been designed to assess association rather than predictive performance. This is appropriate for early-stage biomarker research, but it limits conclusions about clinical utility. Formal analyses of incremental prediction are rare, and among the studies summarized here, only 1 cohort has evaluated whether gestational biomarkers improve discrimination for later clinical CV disease beyond conventional predictors, with marker-specific differences in performance.^22^ This distinction is important because statistical association does not necessarily translate into improved individual-level risk prediction or clinical usefulness. In addition, many cohorts are underpowered for hard CV endpoints, and therefore focus on intermediate outcomes, subclinical phenotypes, or relatively short follow-up. Low event numbers limit the stability of multivariable models, increase the risk of overfitting, and hinder credible estimates of incremental value. Finally, generalizability remains uncertain. Many data sets reflect specific health systems and population structures, and external validation across diverse ancestral, socioeconomic, and clinical contexts is limited. If pregnancy-based risk phenotyping is intended as a scalable strategy, model transportability and equity of performance should be demonstrated.

### Research priorities for translation and implementation

**Figure 3:**
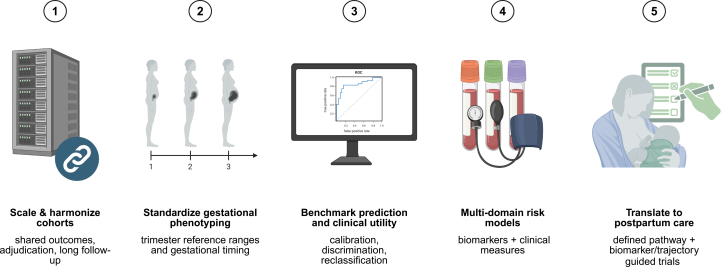
Five Research Priorities for Translating Pregnancy and Antenatal Care Into Opportunities for Cardiovascular Prevention in Women

Moving from promising associations toward clinically actionable prediction and prevention will require a coordinated research and implementation agenda, as outlined in Figure 3.

The first priority is scale and harmonization. Because clinical CV events remain relatively uncommon at the given age, meaningful progress depends on international collaboration that unites large data sets with shared outcome definitions, consistent event adjudication, and sufficiently long follow-up. This should not only be a clinical collaboration between obstetrics and cardiology, but also a scientific collaboration that integrates epidemiology, biostatistics, laboratory medicine, and implementation science to ensure comparable measurements and reproducible analyses.

Second, standardization of gestational phenotyping is essential. Biomarker interpretation in pregnancy requires trimester-specific reference ranges and an explicit treatment of gestational timing. For markers with dynamic trajectories, future work should move beyond single values toward trajectory features or standardized gestational age adjusted scores, analogous to blood pressure trajectory phenotyping. This will improve biological interpretability and comparability across studies.

Third, the field should adopt prediction and clinical utility assessment as a methodological standard. Future studies should prespecify a clinically realistic reference model and quantify whether biomarkers and trajectories add information beyond that baseline. At minimum, evaluation should include calibration, discrimination, and reclassification.

Fourth, progress will likely require multidomain approaches rather than single markers. Multimarker panels spanning angiogenic and cardiac biomarkers should be analyzed alongside both cross-sectional and longitudinal blood pressure features, and complemented by metabolic parameters such as weight and glucose trajectories, to reflect the multifactorial nature of postpartum CV risk. The goal should rather be parsimonious, externally validated models that can be implemented within routine care, rather than increasingly complex signatures that are difficult to reproduce across settings.

Fifth, translation requires an explicit care pathway after risk identification. Pregnancy-based screening becomes meaningful only if it triggers a structured postpartum prevention strategy. Biomarker and trajectory guided interventional trials are therefore a logical next step, testing whether targeted follow-up, blood pressure management, and scalable lifestyle interventions reduce postpartum hypertension and longer-term CV risk. Such studies will also need integrated obstetric CV care models and sustained access to postpartum care. Otherwise, risk identification will not translate into improved outcomes.

## Conclusion

In summary, pregnancy provides a uniquely monitored and physiologically demanding period in which placental, vascular, and myocardial signals can reveal latent CV vulnerability earlier than conventional midlife screening. The available evidence supports the concept that biomarkers and blood pressure patterns measured during pregnancy capture risk beyond binary obstetric outcomes. However, advancing from association to clinically useful prediction and prevention will require large, harmonized cohorts, standardized gestational phenotyping, routine incremental value testing, and integrated care models that connect pregnancy risk signals to effective life-course prevention. Although this review highlights the potential of pregnancy as a CV risk screening window, the current evidence base remains insufficient to support for implementation of antepartum biomarker-based risk stratification into routine clinical practice. Further research into the discriminative and predictive performance of these markers is needed before such a step can be justified.

### Declaration of Generative AI and AI-Assisted Technologies in the Writing Process

Figures were created with BioRender. ChatGPT (GPT-5.2) and Claude (Sonnet 4.6) were used solely for language polishing, including improving sentence structure, clarity and grammar of author-written text. No original content was generated by AI. All ideas, interpretations and conclusions were developed by the authors. The authors take full responsibility for the manuscript content.

## Funding support and author disclosures

Dr Debora Ehrhardt is supported by the Deutsche Forschungsgemeinschaft (DFG, German Research Foundation) - 423813989/GRK2606. Drs Bacmeister and Nolde are supported by the Berta-Ottenstein-Program for Clinician Scientists, Faculty of Medicine, University of Freiburg, Germany. Dr Bacmeister is supported by the “Stiftung zur Förderung der Erforschung der Zivilisationserkrankungen”, Baden-Baden, Germany. Debora Ehrhardt is supported by the Deutsche Forschungsgemeinschaft (DFG, German Research Foundation) - 423813989/GRK2606. All other authors have reported that they have no relationships relevant to the contents of this paper to disclose.
